# Critical components of social prescribing programmes with a focus on older adults - a systematic review

**DOI:** 10.1080/02813432.2023.2237078

**Published:** 2023-07-24

**Authors:** Emil Rapo, Erika Johansson, Frida Jonsson, Åsa Hörnsten, Anna Sofia Lundgren, Ingeborg Nilsson

**Affiliations:** a Department of Community Medicine and Rehabilitation, Umeå University, Umeå, Sweden; b Department of Epidemiology and Global Health, Umeå University, Umeå, Sweden; c Department of Nursing, Umeå Univesity, Umeå, Sweden; d Departmentof Culture and Media Studies, Umeå University, Umeå, Sweden

**Keywords:** Social prescribing, loneliness, older adults, primary healthcare, person-centred care

## Abstract

**Aims:**

The aim of this study was to identify and evaluate critical components within social prescribing programmes that can impact loneliness, health, or well-being among older adults.

**Methods:**

A systematic review with a narrative synthesis was conducted by systematically searching five databases. A total of 1193 hits were identified, screened, and assessed. Twelve studies were included, with data being extracted and deductively analysed in an iterative manner and then tabulated together with outcomes in order to find common narratives.

**Results:**

Three critical components were identified: Assessment before prescription, matching participants with relevant activities, and individualised support from link worker. These critical components seemed important for the success of social prescribing programmes since they had an impact on loneliness, health, and well-being. All together, these results highlight the importance of person-centeredness in the prescribing process.

**Conclusions:**

The three critical components identified may prove useful in further research, evaluation, or implementation of social prescribing programmes. Important aspects for further evaluation are discussed.

## Introduction

Since loneliness, especially among older adults, has been identified as an important public health problem during the last decade [1], the potential consequences of lacking support and feeling isolated has become increasingly apparent during the COVID-19 pandemic [2]. According to previous research, loneliness has been associated with a range of health problems and diseases, illustrating a deteriorating effect on health and well-being [3–6]. McHugh Power et.al [7]. found that loneliness is in a bidirectional relationship with social engagement, which indicates that being engaged in social activities can be an effective way to reduce loneliness [7–9] as well as improve well-being and health [3,5,10]. It seems that these outcomes are common when evaluating interventions targeting loneliness.

Various efforts have been made and interventions implemented to raise awareness about the dangers of loneliness in the UK, USA, Canada, and Denmark [11]. One such intervention is focused on connecting lonely patients from primary healthcare with resources in the community—commonly referred to as social prescribing (SP) [12]. The process of helping lonely patients find and engage in social activities that could contribute to reduced loneliness is often facilitated by a link worker who has an established network of existing contacts within the community. Link worker is a common term used to describe a wide variety of roles within SP programmes that can include assessing patients, but it usually refers to someone helping patients find activities and offering them support [13]. While the body of research exploring and examining the role of SP has grown rapidly during the last decade, the studies that have assessed its effects on loneliness, health, and well-being tend to show inconsistent and sometimes contradictory results [14,15].

One challenge in evaluating SP programmes is having to contend with the variation between them, since SP programmes are heterogeneous due to unique local contexts [16]. Despite the differences, a range of positive outcomes for individuals, such as reduced loneliness, improved mood, improved mental well-being, and a reduction in healthcare use, have been reported [17,18]. These results should be viewed with some caution since evaluations have been critiqued for their low quality, as they often have small sample sizes and lack control groups [19,20]. There is a consensus regarding the need for further standardisation of SP programmes and the development of common concepts [14,21].

An initial attempt to develop, implement, and evaluate Social Prescribing in Sweden (SPiS) is currently underway [22]. When developing complex programmes such as SPiS, it is important to outline the programme architecture in a systematic way by identifying critical components, i.e. both formal and informal strategies that impact loneliness, health, and well-being in existing programmes. This knowledge could then be used as a basis for understanding the critical components that should be included in SP programmes in order to reduce loneliness and improve health and well-being.

### Aim

The aim of this study was to identify and evaluate critical components within social prescribing programmes that can impact loneliness, health, or well-being among older adults.

## Material and methods

A systematic literature review with a narrative synthesis was conducted to identify critical components of existing SP programmes. This study followed the PRISMA guidelines [23] and is described in a protocol as part of a larger project [22].

### Search strategy

An exhaustive systematic search strategy was devised by the authors with input from a research librarian. The literature search was conducted during the month of March 2020 using PubMed, Medline, PsychINFO, CINAHL, and SOCIndex. The aim of the search strategy was to assemble a collection of all published studies that evaluated SP with regard to loneliness, health, and well-being. Test searches guided the final search strategy, and the researchers did not find any other alternative names or terms for SP. The final search included ‘social prescribing‘ OR ‘social prescription‘ OR ‘community referral‘ OR ‘community linkage‘ OR ‘community connection‘ in titles or abstracts. The complete search is available in Supplementary Appendix 1.

### Selection of studies

**Figure 1.**
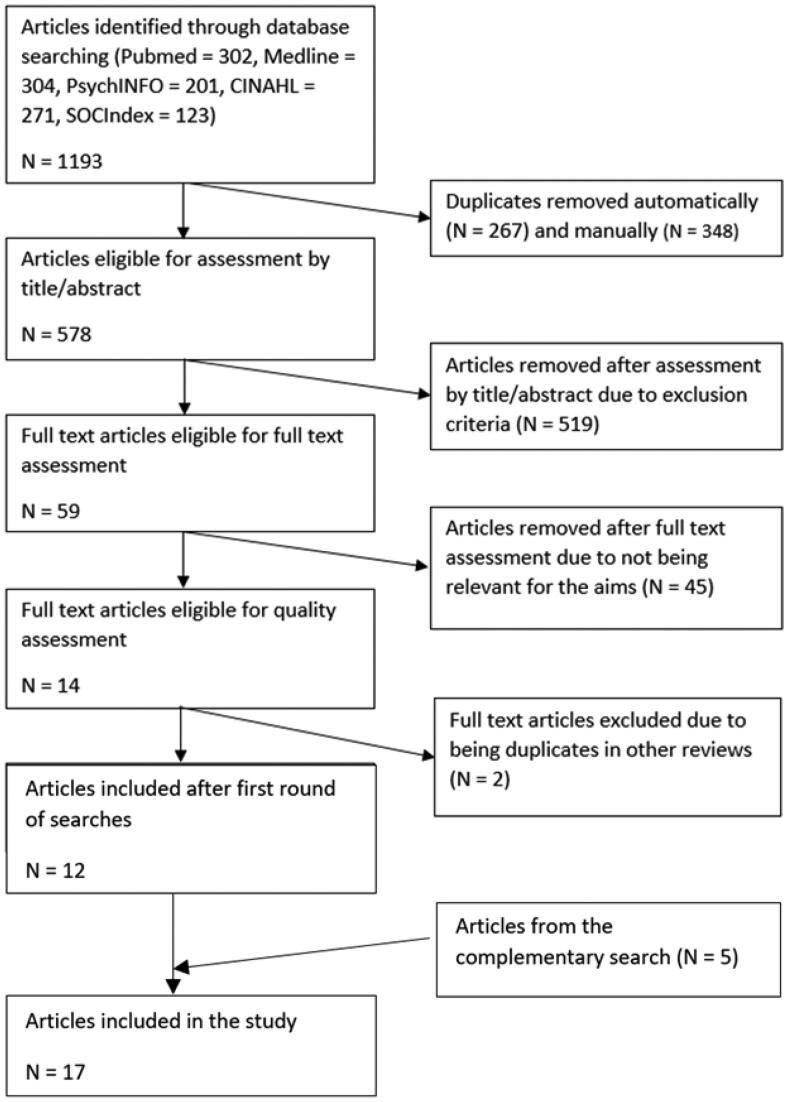
Prisma flowchart.

The studies were assessed by ER and EJ using 2020 Rayyan Systems software [24]. Initially, all articles were independently assessed. Thereafter, divergences were discussed with the third author IN until a consensus was reached. The assessment was performed in two stages, firstly by title and abstract, and secondly by reading the full texts. See Figure 1 for the search strategy and outcomes. In order to be included in the assessment, articles had to evaluate SP either in a qualitative, quantitative, or mixed-methods manner. Articles were excluded if they did not include older adults or if they did not focus on loneliness, health, or well-being as an outcome.

### Quality assessment

The included articles were assessed qualitatively using four different tools. These were the SBU (Swedish Agency for Health Technology Assessment and Assessment of Social Services) tool for qualitative studies [25], the SBU tool for quantitative non-randomised studies [25], the Mixed Methods Appraisal Tool [26], and the AMSTAR-2 [27] tool for systematic reviews. The appraisal was performed by ER, with IN and ÅH also reviewing three studies each in order to achieve consensus.

### Data extraction

Data were extracted from the included articles by ER, organized into a preliminary table by ER, EJ, and IN, and then further discussed and revised with input and support from all the authors. Based on these discussions, the extracted data were organized into two separate tables, one with descriptive data of the main characteristics (Table 1), and one including the critical components and outcomes identified (Table 2). The data were condensed, and language was harmonised for readability. The main principle adopted while developing the tables was to stay as close as possible to the original texts from the articles. In cases where different synonymous terms were used, the team of authors agreed upon a synchronised terminology (e.g. ‘link worker’).

**Table 1.** Descriptive table.

Study	Method	Context	Participants	Outcome measures	Quality assessment	Attrition/ Adherence
Bickerdike et al. [28]	Systematic review. Nine databases were searched from 2000–2016 and analysed with narrative synthesis. 15 evaluations were included with varied methods and many forms of SP included.	All evaluations were from the UK.	No information reported	Warwick-Edinburgh Mental Well-being Scale (WEMWBS), Hospital Anxiety and Depression Scale (HADS), General Anxiety Disorder-7 (GAD-7), Patient Health Questionnaire-9 (PHQ-9), Clinical Outcomes in Routine Evaluation-Outcome Measure (CORE-OM), Work and Social Adjustment Scale (WSAS), General Health Questionnaire (GHQ-12), Functional health with COOP/WONCA and some bespoke measuring tools. Any measurement or reporting of attendance. Any reporting of costs. Qualitative evaluations of patient experience and referrer experience.	Low risk of bias	Attrition rates were not reported. Adherence to initial SP appointment ranged from 50–79% in the included studies. Adherence to social activity after referral ranged from 58–100% in the included studies.
Kilgarriff-Foster and O’Cathain [29]	Literature review. 5 databases were searched and results analysed by narrative synthesis. 24 evaluations with varied methods and many forms of SP included.	All evaluations were from the UK.	The majority of participants were over 40 years old and female. Causes for referral was isolation due to unemployment or frequent GP attendees with inexplicable symptoms.	Any measures of health or well-being such as Anxiety and depression (HADS), Mental Well-Being (WEMWBS), General health (GHQ-12). Any other measures of impact. Qualitative evaluation of participant experience.	Medium risk of bias	Attrition rates not reported. Adherence to social activity only reported in one included study, 58%.
Pescheny et al. [30]	Systematic review. 11 databases were searched with relevant search terms and results analysed with narrative synthesis. 16 evaluations with varied methods and many different forms of SP programmes included.	All evaluations were from the UK.	Participants were described as older in five studies, referrals were for a mix of long-term conditions, frequent attenders of primary health care, psychosocial needs, and in five studies loneliness or social isolation.	Mental Well-Being (WEMWBS), Anxiety and depression (HADS), Measure Yourself Medical Outcome Profile (MYMOP), General health (GHQ-12), Functional health (COOP/WONCA), Friendship Scale. Qualitative evaluation of patient experience.	Low risk of bias	Attrition rates not reported. Adherence rates not reported.
Chatterjee et al. [31]	Systematised literature review. 12 databases were searched with relevant search terms. 40 evaluations were included, nine were SP and the rest a mix of other schemes.	All evaluations were from the UK.	11 studies report including older adults. Majority of participants were included for some underlying or previous health issue.	Anxiety (GAD-7), Cost effectiveness (Quality Adjusted Life Years), Depression (PHQ-9), Functional Health (CO-OP/WONCA), Hospital admission (Hospital Episode Statistics HES) Mental health (GHQ), Mental well-being (WEMWBS), Physical activity (Timed Up and Go test), Psychological well-being (HADS), Quality of life (Delighted-Terrible faces), Social isolation (Social isolation), Social support (Duke-UNC Functional Social Support Questionnaire) Qualitative evaluations of patient and professional experience.	Medium risk of bias	Attrition rates not reported. Adherence rates not reported.
Elston et al. [32]	12-month longitudinal pretest /posttest study. Questionnaire data were collected by healthcare staff from 126 participants during first and last meeting, healthcare use data were collected from IT systems.	Devon, UK, a rural area with a proportionally older population with some pockets of deprived areas.	Participants were over 50 years of age, 73% were female with two or more long-term conditions and many having assisted forms of living.	Well-being (Well-being Star), Activity (Patient Activation Measure), Mental Health & Well-being (Warwick-Edinburg Mental Well-Being Scale), Frailty (Rockwood Clinical Frailty Scale), health & social care use, health and social care costs.	Low risk of bias	151 participants were referred to SP, 126 completed 12-week assessment, 86 completed full 12-month data. Adherence rates not reported.
Morton et al. [33]	Pretest /posttest study. 136 participants filled out questionnaires at the beginning and end of their course.	Fife, UK	Participants were of an average age of 52 with older adults included, and many with ongoing mental health intervention/medication.	Anxiety and depression (Hospital Anxiety & Depression scale), Self-efficacy (General Self-Efficacy scale), Mental well-being (WEBWBS) Anonymous feedback	Medium risk of bias	Attrition rates not reported. Adherence rates not reported.
Pescheny et al. [34]	Pretest/ posttest study. Link workers collected data at first and last appointments from 63 participants.	Urban UK setting described as having a large ethnic minority with higher than average multiple deprivation.	Participants were of all ages, between 24 and 83, with a female majority and a majority outside employment.	Mental well-being (WEMWBS)	Medium risk of bias	84.8% were lost to follow-up or did not engage after initial assessment. Adherence rates not reported.
Kellezi et al. [35]	Mixed methods. Questionnaires were administered by healthcare workers at baseline (*n* = 630) and after approx. 4 months (*n* = 178). Semistructured interviews with 35 patients and staff, thematic analysis applied.	East Midlands, UK	Participants were from all ages, with an average age of 60.4 years. The SP programme was focused on people suffering from loneliness and chronic illness.	Questionnaires: social groups (custom), community belonging (custom), loneliness (UCLA loneliness scale), Health service use (custom). Interviews: Participant experience of social connection and effects of SP programme.	Low risk of bias	Of the 630 participants, 178 participated in follow-up. Adherence rates not reported.
Woodall et al. [36]	Mixed methods. Questionnaires were administered by link workers at first and last meetings, approx. six weeks, for 342 participants. 26 participants were interviewed and 17 link workers were recruited for a focus group discussion. These were analysed thematically.	A large city in northern England, UK	Participants were of a mean age of 53 years, ranging from 19–94, and a majority was female.	Questionnaires: well-being (WEMWBS), mental and physical health (EuroQol-5 Dimensions), social isolation and loneliness as well as the ability to manage long-term conditions (Campaign to End Loneliness Measurement). Interviews: Experiences and outcomes, what works for whom and why, perspectives on the service.	Low risk of bias	Attrition rates not reported. During this period, SP had 2250–3750 referrals, and they comment that the vast majority were lost to follow-up or did not engage in SP.
Heijnders et al. [37]	Interview study. Ten participants were purposefully chosen by link workers to include a variety of experiences from the programme. Results were analysed thematically.	Netherlands	Participants had a mean age of 69, withan even gender split. Patients had some psychosocial issue that was not explained by a medical condition.	What happens in the social prescription process? What changes do people experience regarding social participation?	Medium risk of bias	Several participants had reduced activity attendance.
Payne et al. [38]	Interview study with a realist perspective. 17 participants were purposefully sampled from the programme. Semistructured interviews analysed by phenomenological analysis.	Sheffield, UK, that is described as a highly deprived area according to postcodes.	Participants were between 45–84 years old, majority female, mostly British. Many were referred for mental health reasons or for improving physical health.	Identify how participants perceived any benefits from SP. Emerging themes were tested against published qualitative findings.	Low risk of bias	Several participants had reduced progression through the programme.
Wildman et al. [39]	Interview study. Twenty-four participants with a variation of experiences were recruited. Semistructured interviews were analysed from a grounded theory perspective.	Newcastle upon Tyne, UK, described as an inner-city area with high socioeconomic deprivation	Participants were aged 40 to 74, and there was an even gender split, with mental health issues and multimorbidity being common.	Service users perspectives on link worker SP, with a focus on emergent narratives.	Low risk of bias	Several participants had reduced attendance to social activities.
Bild 2022 [40]	Systematic review. Four databases were searched from 2009–2019 and analysed with narrative synthesis. 77 articles were included with varied methods and many forms of SP included.	Not reported	The mean age of participants in each study was 50, with most included articles having a focus on older adults.	Any measures or qualitative results were described in themes of social connection; improvement in management of health and health status; improvement in mental health and wellbeing; life enrichment; and link worker support.	Low risk of bias	Attrition rates not reported. Adherence rates not reported. Lack of adherence measures in evaluations was reported as an issue.
Foster 2020 [41]	Mixed methods. Qualitative interviews with service-users, volunteers and link workers and quantitative analysis of routinely collected data from 2017–2019 as well as additional data collected at 3 month follow-up.	The study included programs from a wide range of setting within the UK	The sample size was 10643 with 66% being female, 70% being white british, 65% lived alone, 80% were over 50 years of age, 50% experiencing health issues and 25% experiencing mobility issues.	Loneliness measured by UCLA Loneliness scale Interviews focused on the experience and impact of the support, service delivery and sustainability.	Low risk of bias	Attrition rates not reported, issues with collecting data mentioned. Adherence rates not reported.
Kiely 2022 [42]	Systematic review. Eleven databases were searched up to 2021 and analysed with narrative synthesis. 8 articles were included with varied methods and many forms of SP included.	Primary health care and community contexts within the UK and USA. Two articles specifically mention being in a economically deprived area.	6500 participants across the articles, majority female, age range from 29 − 75.	Primary: Health-related quality of life (HRQoL), as measured by a validated instrument (EQ-5D, SF-12). Mental health outcomes, as measured by a validated instrument (HADS-A) for screening for mental health conditions. Secondary: Patient-reported outcomes on social-connectedness or isolation, self-rated health, patient experience of care, treatment burden, self-management behaviour and self-efficacy. Physical activity and function included measures of physical activity (self-reported or objectively measured), physical function, activities of daily living. Health service utilisation measured *via* primary care or hospital records or self-reported. Any physical health data reported and any cost data or social return on investment data.	Low risk of bias	Attrition rates not reported. Adherence rates not reported.
Kim 2020 [43]	Pretest/ posttest study. Using the PRECEDE-PROCEED method, 10 participants were followed over a 10-week period.	Rural area within South Korea.	10 participants, all female, mean age of 84, all had atleast one chronic disease, 7 participants were illiterate, 9 were living alone.	Depression (GDS-Korean), Loneliness (UCLA-Loneliness scale), Social participation attitude, Self-efficacy (GSE), Self-esteem (Rosenberg self-esteem scale)	Medium risk of bias	16 participants, of which 10 participated the full 10 weeks and were included in the study. All 10 adhered to atleast one activity, with some adhering to all three program activities.
Reinhardt 2021 [44]	Systematic review. Nine databases were searched from 2000 to 2019 and nine articles were synthesised, with highly heterogeneous studies and social prescribing programs.	All included studies were from the UK	12359 participants plus approx. 9000 in one study. Participants age range from 16–85.	Loneliness (UCLA-loneliness scale, Adult Social Scale and Public Health Outcome, Hawthorne friendship scale)	Low risk of bias	Attrition only reported in one included article, from 254 at pretest to 215 at posttest. Adherence not reported.

**Table 2.** Results table.

Components Studies	Assessment before prescription	Matching participants with activities	Individualised support from link workers	Outcomes
Bickerdike et al. [28] Systematic review	In all studies, link worker met with participants to discuss their needs.	Focus on a wide range of community activities. Patient-centred focus not reported.	Link worker (LW) support in general was not reported. Some studies reported link workers having good local networks. Participants could receive support with navigating welfare programmes.5 of 15 studies reported LW training, 7 did not have training, 3 did not report.	Health and well-being generally improved in quantitative measures, but authors recommend caution due to poor quality of included studies. Patient experiences reflected reduced loneliness and social isolation, improvement in mental and physical health. Experiences also raised the importance of confidence, successful matching, overcoming barriers, informed referral, and clear communication between actors.
Kilgarriff-Foster and O’Cathain [29] Systematic review	One study described 40–90-minute appointment to identify needs and appropriate activities. Assessment was not reported in other studies.	Focus on a wide range of community activities, with one study described finding appropriate activities, but no other patient-centred focus reported.	LW support was in-person meetings. One study described LW accompanying patients to activities, with patient receiving increased support early. Another study described a small number of follow-up appointments, average was two. LW received training in all included studies.	Reduced anxiety (*p* < 0.002), improvement in well-being, reduction in symptoms and attaining goals, but authors recommended caution due to poor quality. Patient experience highlighted self-efficacy. Too long wait times resulted in patient not engaging.
Pescheny et al. [30] Systematic review	All included studies had individual assessment to identify non-medical needs of service users, sometimes using tools or motivational interviewing.	Focus on a wide range of community activities, with most studies describing some form of patient-centred focus.	Link worker support was in-person meetings in all studies. Support was described as personalized and continuous. LW accompanied patients to activity in one study, two studies described increased support early with a patient-centred focus. Participants could receive support with navigating welfare programmes.LW training was partially reported.	No significant results for mental health. Results for well-being, general health, function, and loneliness was mixed. Patients also felt reduced social isolation in qualitative studies. Patient experience highlighted support from link workers as central for behavioural change, attendance, building confidence, and overcoming barriers in their daily lives. Mastering skills made participants continue.
Chatterjee et al. [31] Systematised review	Not reported	Partial focus on a wide range of community activities. Patient-centred focus not reported	LW support was in-person meetings. Results include 3 studies with ‘supported referral‘ that was described as helping overcome barriers and offering moral support depending on need. Signposting schemes could offer guidance to welfare programmes.LW training was partially reported.	Reduced anxiety, depression, loneliness, and social isolation. Improved mental well-being and physical health. Improved confidence and self-efficacy. Link workers with local knowledge an important facilitator, with limited choices and lack of economical support important barriers. No statistical results were given, quantitative and qualitative outcomes were reported together, and authors recommended caution due to poor quality.
Elston et al. [32] 12-month pretest/ posttest	Initial 30 min meeting to determine light or ‘holistic‘ approach. Holistic approach consisted of using multiple tools over several meetings.	Focus on a wide range of community activities, with a focus on understanding what matters to participants and setting goals for living well.	LW support was in-person and by phone or home visits when needed. Support consisted of coaching and practical support over 12 weeks, also support for navigating welfare systems. LW received training in goal-setting, using tools, using a strength-based focus, co-producing a plan, and managing risk. Key skills included listening skills, emotional support, advice, practical assistance, and coaching.	Improved mental well-being (*p* = 0.000), well-being (*p* = 0.000), patient activation (45/81, 55.6%), reduced frailty (4.6%). Link worker improved continuity of care and offered support for both patients and carers during difficult times. Some patients had sudden deteriorating health that affected outcomes.
Morton et al. [33] Pretest/ posttest	Not reported	Only a few courses offered as part of the SP programme (Meditation, Painting, Photography, Jewellery, Arts & Crafts, and Pottery). Patient-centred focus not reported	LW support was in-person meetings as part of the courses offered. Participants received no support outside courses.LW received training in working with mental health, such as identifying and supporting someone with anxiety.	Reduction in depression and anxiety (*p* < 0.001), improved mental well-being (*p* < 0.001), and improved self-efficacy (*p* < 0.001). Majority of participants also received therapy or medical treatment during the intervention.
Kellezi et al. [35] Mixed methods	Initial one-hour needs assessment with healthcare personnel who then referred to LW.	Focus on a wide range of community activities, with a patient-centred focus	LW support was in-person meetings over 8 weeks, with programme length dependent on the pathway. LW regularly checked participant’s progress and could accompany participant to first activity if needed. LW training not reported	Feelings of group membership increased (*p* = 0,022), which was a positive predictor for community belonging (*p* = 0.01), which in turn was a negative predictor for loneliness (*p* = 0.0001) that positively predicted reduced primary healthcare use (*p* = 0.002).GP experience reflected need for holistic interventions, and that in the current medical model GPs exacerbate rather than help with poor social health. LW experience reflected on patients not knowing what was around them and the importance of reconnecting with the community. Patient experience positively noted the increased amount of time they had to discuss their problems and the tailored, encouraging support they received. Patients reported increased confidence and described LW support as vital, especially when the LW accompanied them to their first activity, where being positively welcomed was crucial. The LW support was essential in connecting with others and sustaining meaningful connections in groups. SP could help others besides the patient, causing a ‘ripple effect‘. Important to have a feedback loop.
Woodall et al. [36] Mixed method	Needs assessment could be short and over the phone, or longer with in-person meetings.	Focus on a wide range of community activities, with a focus on exploring social support needs.	LW support was in-person meetings and by phone when needed. Target limit was 6 sessions, but many exited within 16 weeks, with an explicit statement to avoid participants becoming dependent on the service. The SP service did not offer much support, but activities could be very supportive. Could offer finance/debt adviceLW received training.	Improved well-being (*p* < 0.001) with a negative relationship with age (*p* < 0.001), especially under age 50 (*p* = 0.02). This was supported by interviews. Reduced depression & anxiety (*p* < 0.001), improved self-rated health (*p* < 0.001), and small improvement to social network scores (*p* < 0.001, *d* = 0.35). Interviews reflected increased social connectedness due to finding good matches between patients and activities. This led to increased feelings of confidence and purpose. Sharing experiences in social settings, having skilled link workers, flexible support, and a thriving community were seen as important for success, particularly in engaging men.
Pescheny et al. [34] Before/after study	Individual assessment with motivational interviewing to identify non-medical needs.	Focus on a wide range of community activities. Patient-centred focus not reported.	LW support was in-person meetings, with support tailored according to person’s needs and continuous personalised support offered.LW training not reported.	Statistically improved mental well-being (*p* < 0.0001), but the results were not clinically significant. The authors reported LW turnover, fragmented service delivery, language barriers, and difficulties in completing questionnaire as possible reasons for the high loss to follow-up.
Payne et al. [38] Interview study	Participants were triaged by phone or in person.	Focus on a wide range of community activities, with a focus on personalised linking to activities.	LW support was in-person and by phone, with LW accompanying participants to activities when needed. Programme length was between six months and five years.LW training not reported.	Improved confidence the central positive outcome in all themes. Five central themes for a successful intervention were discussed; **Receiving professional support** to overcome practical barriers and improving confidence, **Engagement with others** in purposeful and enjoyable activities that provided routine, **Learning new skills** improved confidence and reinforced a shared experience, **Changing perceptions** provided improved confidence and an appreciation of personal strengths, and **Developing a positive outlook,** where improved confidence and independence motivated participants to pursue new goals and have optimism for the future. Other healthcare needs were an important reported barrier for participation among those that did not progress as expected.
Heijnders et al. [37] Interview study	Intake session with strengths-based approach to evaluate participant’s life holistically in order to find sources of positive strength and also possible barriers to participation.	Focus on a wide range of community activities, with a step-by-step approach that focused on what the participant enjoys doing, and with encouragement to choose activities that promote social health.	LW support was in-person meetings and by phone, or home visits when needed. LW also registered for activities together with the participant and monitored progress by phone. LW received training.	The authors summarized outcomes in which participants mostly felt healthier, became more self-reliant, and regained perspective and control over their lives. Five themes emerged in the study: **Life events** highlighted the sudden changes that caused the participants current situation, such as loss of spouse or retirement. **Referral and intake process** highlighted the major obstacle of starting something new alone, and the personalised service with follow-ups encouraged adherence. **Strength and responsibility** highlighted the importance of activities reflecting participants needs, interests, and hobbies as well as finding their own solutions to problems. **Self-reliance** highlighted the need for strong incentives and support from the LW and the community for continued participation. **Social activation** also highlighted the importance of matching interests and connecting with others.
Wildman et al. [39] Interview study	Evaluation by motivational interviewing & proprietary tool to find life areas that the participant wanted to improve.	Focus on a wide range of community activities, with LW helping patients identify personalised and achievable goals.	LW support was in-person meetings and by phone, or home visits when needed. Programme length was on average two years, with option to go longer. LW had regular check-ups to monitor progress towards participants own goals. LW would accompany participant to activity when needed. Programme could address housing, debt, and welfare needs. LW received training in behavioural change methods.	Themes that emerged were**: Importance of the LW-participant relationship** with the personalised approach seen positively. The LW was essential in building confidence as was their wide knowledge of community services. **Making and maintaining progress and long-term condition management** highlighted the participant’s growing confidence as central for improvement, and the long-term focus of the programme facilitated continued self-regulation. **Setbacks and barriers** highlighted the difficulty of maintaining change over time, with health-related problems being the most common cause of relapse, or physical barriers such as lengthy or costly travel, unsuitable scheduling, an unsafe location, language barriers, or cultural inappropriateness. LW turnover and poor continuity was also reported as detrimental. **Fluctuating levels of engagement** highlighted that contact with SP service declined naturally as participants engaged in activities, but for some, the two-year time constraint was seen as too short, and they needed much more long-term support.
Bild 2022 [40] Systematic review	Programs included a wide range of different assessments as well as no assessments at all.	Most included programs had a focus on specific activities, such as arts & crafs or walking groups. Some programs had a focus on a wide range of community activities.	LW support included a wide range, from ‘group leaders‘ to trained LW-professionals. Some programs were open ended, with others having a clear structure in time and amount of LW-meetings. LW training varied significantly in the different programs.	Improved social connection, including a sense of group connection and reduced feelings of loneliness. Improved mental health and wellbeing, although authors recommend caution as there is some indication that those with poor mental health and wellbeing are not always included in the results of evaluations due to attrition/adherence. Increased physical activity, increased sense of purpose, independence, and confidence due to new meaningful activities.
Foster 2020 [41] Mixed method	Link-workers and volunteers performed an needs assessment.	Focus on developing confidence in order for participants to access a wide range of community activities with a person-centred focus.	LW support amount and length was tailored according to individual’s needs, with volunteers offering further support such as following participants to activities.	Reduced loneliness, favourable return on investment. Qualitative experiences reflect improved self-esteem, confidence and wellbeing.
Kiely 2022 [42] Systematic review	The programs describe face-to-face needs assessment by link-worker, action planning and motivational interviewing, mapping resources,	Referral activities were tailored to the individual with a focus on a wide range of community activities.	Link-worker support varied from 1 meeting to 2 years of ongoing support, with specific type of support not reported.	No evidence for improved health-related quality of life or mental health. no evidence for effectiveness in improving social support, physical function and activities, or primary healthcare utilisation, though there was a suggestion from two studies that interventions led to improved self-rated health and two others reported higher patient ratings for quality care. The certainty of the evidence is low or very low overall due to risk of bias, heterogeneity among studies, inconsistency and imprecision.
Kim 2020 [43] Pretest/posttest	Face-to face health evaluation with public health doctor	Referral activities were a music group, a self-help group and a gardening group.	Coordinators and volunteers held the program activities, level of support in our outside programs was not reported.	Reduced loneliness and depression, improved social participation attitude and self-esteem.
Reinhardt 2021 [44] Systematic review	Assessment not clearly reported	Included articles ranged from a focus on individually tailored community activities to a more specific activity such as prescribed museums.	Support varied from simple signposting to long-term individualised support from link-workers.	Reduced loneliness and use of services especially among those aged under 60.

### Data synthesis and analysis

Due to the heterogeneous nature of the data, a narrative synthesis was applied that included four steps, developing a theory, developing preliminary synthesis, exploring relationships in the data, assessing the robustness of the synthesis [45]. The included articles were thus read thoroughly, a theoretical model was developed by the research team, and any descriptions of the programmes were extracted verbatim from the background, results, and discussions in the articles in order to develop a preliminary synthesis. These data were analysed according to content by identifying differences and similarities in the data and organizing them into descriptive themes according to preliminary components. These were then discussed and refined by the whole research group, which included expertise in occupational therapy, nursing, epidemiology and ethnology, in an iterative process in order to identify analytic themes beyond the primary descriptive themes. The three remaining themes were then tabulated together with outcomes in an attempt to explore relationships between the critical components and outcomes in Table 2. The synthesis was evaluated for robustness by including quality assessments.

## Results

The initial search resulted in 1193 articles, which are presented in the flowchart in Figure 1. Duplicates were removed automatically (*n* = 267) and manually (*n* = 348), thus leaving 578 articles. These articles were individually and blindly assessed for relevance by ER and EJ by reading titles and abstracts, which left 59 articles. These were individually and blindly assessed (ER and EJ) for relevance by reading the full text of the article. Fourteen articles were deemed relevant, and they were subsequently assessed using quality assessment tools. Two empirical articles were later excluded since their findings were part of the results of systematic reviews already included in the study. At the end of this process, 12 articles remained and were included in the study.

### Sample characteristics

The sample of 12 articles [28–39] provided the empirical material for our analysis. All the articles had an explicit aim to evaluate one or multiple SP programmes with regards to loneliness, health, or well-being outcomes. Table 1 presents a summary of their characteristics. The included articles, which were authored in the United Kingdom (*n* = 11) and Netherlands (*n* = 1), used a variation of methods and approaches. Four were systematic review studies with a narrative synthesis [28–31], three were longitudinal studies with baseline and follow-up measurements [32–34], three were qualitative interview studies [37–39], and two were mixed methods studies [35,36]. Three studies were described as being conducted in urban settings [34,36,39], and one in a rural setting [32]; the other studies did not define their geographical context. The sociodemographic context in which the SP programme was situated was described as socioeconomically deprived in four articles [32,34,38,39]; the other studies did not include descriptions of the context. All the studies included patients with some form of long-term condition, poor physical or mental health, frequent healthcare visits, or having experienced a recent life event such as loss of spouse, unemployment, or retirement. Three studies [32,34,35] reported information about attrition rates of participants in the studies, while six studies [28,29,36–39] included descriptions on adherence to the SP-programme.

### Identified critical components

The narrative synthesis revealed three components in SP programmes that did have an impact on loneliness, health, or well-being in older adults: *assessment before prescription*, *matching participants with activities,* and *support from link workers*. Table 2 gives an overview of the components and outcomes in the included studies. Below, we first report the critical components identified, and then we describe whether, and how, these critical components were of importance for specific outcomes.

### Assessment before prescription

Assessment before prescription was reported as a component in many SP programmes. In this step, healthcare personnel assessed participants’ health, personal interests, and living situation. Some studies [30,32,34,37,39] reported the use of tools or strategies such as motivational interviewing, with the main goal of both gathering information about, and building a relationship with, the participant. Elston et al. [32] and Payne et al. [38] reported this step as more of a triage process in order to determine if participants should be referred to a shorter or extended version of the programme. The importance of receiving time and attention to discuss people’s situation before prescribing an activity was highlighted as important [35].

Assessment before prescription was reported in ten studies. Loneliness or social isolation was reduced in three of those studies [28,35,36], and health and well-being was improved in seven of those studies [28–30,32,34,36–39].

### Matching participants with activities

Matching participants with activities was a component that involved the task of link workers attempting to find suitable activities for participants. Six studies [30,32,35,37–39] specifically stated that the matching had to be person-centred or personalised. To facilitate this, the link workers used information gained during the initial assessment, but they also needed knowledge regarding the available activities to choose from. One study [33] stood out as they offered a selection of courses at the healthcare centre as a part of the SP initiative. Eleven studies [28–32,34–39] reported a wide focus on different activities in order to find suitable matches for everyone. They also recommended activities that already existed in the community.

Of the studies (*n* = 11) that reported a focus on a wide range of different activities in the local community when matching activities to the participants, loneliness and social isolation were reduced in four [30,31,35,36] while health and well-being were improved in eleven [28–32,34–39]. The findings underscored that a successful match was characterized by the activity being meaningful, interesting, and available to the participants [28], and that a good match seemed to increase social connectedness and confidence among the participants [36]. Improved confidence and self-efficacy were in general seen as important factors for successful outcomes [28–31,33,35–39]. Successful matching was also dependent on a community with many available activities [35,36]. However, link workers needed to have good knowledge of the available activities in the local community in order to facilitate good matches [31] since the participants often lacked this knowledge themselves [35].

### Individualised support from link worker

The third critical component, individualised support from link worker, included a wide range of support, help, or guidance that was offered after the participant had been matched with an activity. Support from the link worker varied due to the individual needs of the participants, as reported in four studies [35–39]. According to two studies [38,39], the participant’s confidence was boosted when a trustful partnership was developed with the link worker. Furthermore, having a link worker accompany participants to their first activity was regarded as vital for a successful initial participation according to one study [30].

The importance of receiving support from link workers to ensure continued participation in activities was reported in three studies that measured reduced loneliness [31,35,36] and ten studies that measured improved health and well-being [29–32,34–39]. Examples of how link workers supported the participants included accompanying them to activities [30,35] and helping them navigate the bureaucratic welfare systems [28,36].

The findings highlight the importance of support in overcoming barriers such as low self-confidence, and economic or geographical barriers [28–32,34,36,39]. Two studies especially reported on the importance of individualised, flexible support from skilled link workers [35,36], and the importance of individualised support from link workers in managing long-term conditions and other health-related barriers to participation was specifically reported on in four studies [30,32,38,39]. Individualised support from link workers was reported as key for successfully initiating participation in matched activities [28,35,36], continued adherence [30,35,36], as well as mastering skills [30], having an informed referral, and clear communication between actors [28].

### Outcome measures and effects

All the included studies reported an impact on loneliness, health, or well-being, and they are summarized below. Although components and outcomes are described in the different studies, it was not possible to describe reliable patterns between components and outcomes due to the variation in design, methods, and outcome measures. However, of the five studies that reported on loneliness or social isolation as an outcome measure [28,30,31,35,36], three studies reported reduced loneliness [28,31,35], one reported mixed results on loneliness and social isolation [30], and one study reported reduced social isolation [36].

With regard to the improved health and well-being outcome measures, improvement in physical health [28], self-rated physical health [28,30], self-rated mental health [28,30], well-being [28–32,36], self-rated mental well-being [31–34], management of psychosocial needs [29], activation (PAM) scores [32], and reduced depression and anxiety scores [31,33,36] were reported. Eight studies link experiences of improved confidence and self-efficacy from SP to improved health and well-being outcomes [28,29,31,35–39]. No included study reported any negative results e.g. lower level of health or well-being.

## Discussion

Three critical components were identified that aimed to generate an impact on loneliness, health, and well-being: *assessment before referral*, *matching participants with activities*, and *individualised support from link workers*. The various SP programmes differed in how these components were implemented, and the relationship between components and outcomes were often not completely clear. Even though the components within the programmes were often presented as interconnected, we will discuss them below separately for the sake of clarity.

### Assessment before referral

Assessment before referral was a common but diverse practice varying between a triage in order to prioritize other healthcare needs and a comprehensive evaluation of a participant’s needs, wants, and resources. As reported, it was important for the personnel doing the assessment to build trust with participants as well as manage their expectations. The building of trust is essential for primary healthcare providers in general as it affects both patient adherence and outcomes [46]. Performing a comprehensive or ‘holistic‘ assessment also requires that healthcare personnel prioritize time to explore the participants’ individual needs and situation; methods such as motivational interviewing were reported as suitable complements to the assessment process. Since loneliness is a complex phenomenon with many dimensions, there is a high risk of mistaking loneliness for other conditions such as depression [11], further complicating the assessment before referral. Building a relationship with patients is the basis of person-centred care that includes occupational therapy and nursing, and it has also a bearing in social prescribing programmes [47–49]

### Matching participants with activities

When matching participants with activities, information gained from the assessment is compared with available activities in the community, and a suitable activity is decided upon together with the participant. This requires the link worker to understand what demands the activity or environment places on the participant [48]; for example, an outdoors activity might be difficult if the participant has mobility issues, a coffee shop may be challenging for someone who has impaired hearing, or an activity may have an attached cost that is prohibitive. In order to reduce loneliness, a key aspect in matching was for participants to experience the activities as meaningful. Hammel [50] stated that a meaningful activity has inherent meaning for the individual and that it is fulfilling a purpose, i.e. engaging in meaningful activities is a core quality of life. Furthermore, according to Willock [51], in order to perceive a social activity as meaningful, an individual needs to have a sense of belonging and a sense of being a part of a larger whole. This means that a link worker, in order to find an optimal match, needs to navigate and negotiate complex aspects of individual wants and needs, as well as manage many other aspects regarding the environment and the activities together with the participants.

### Individualised support from link workers

Individualised support from link workers was reported in the data as anything the link worker or other professionals do after participants receive their referral to an activity. Support was often given by phone, but it could also include physical support such as accompanying participants to their first activity. The amount of support offered may be individualized depending on the needs of the participants, and support when attending the first activity has been seen as necessary in other evaluations of SP programmes as well [52]. Individualized support during intervention has previously been reported as necessary [53] since needs are not uniform among participants. As different activities and environments place demands on the participants [48], the link worker also needs to be able to identify barriers and help participants overcome them [54]. The included studies described many different barriers impeding participation in social activities, but a frequently reported barrier that participants needed support to overcome was their health status since many also suffer from chronic illness, disabilities, and comorbidities. We can thus infer that an adequate assessment from a competent link worker is important because removing or mitigating health-related barriers seems to significantly affect the success or failure of a social prescription.

### Person-centredness as a unifying theme in the data

All three critical components were strongly characterized by elements of person-centredness, which implies that a standardized procedure meant for broader implementation needs to strike a balance between allowing room for individual tailoring while still following the core aspects of SP. Other studies [14] have discussed the difficulty of evaluating SP programmes due to a lack of standardisation and the differences between programmes.

Person-centredness is a core aspect in healthcare professions such as occupational theory [51] and nursing [55,56]. Person-centred care does come with several challenges, such as having competent personnel that can manage a broad or ‘holistic‘ assessment, or avoiding information overload in patients and the risk of transferring responsibility from healthcare to patients [55], but it is also an ethical demand. For SP programmes, this might signify an increased risk that information gathered during assessments is too personal and sensitive, such as information pertaining to a difficult family situation that requires skill and resources to manage. There is also an ethical challenge and a tangible risk that participants will feel increased pressure to follow the prescription in order to receive further care, or that failing to adhere to the prescription is a personal failure rather than a failure of a healthcare programme, especially if the amount of support from link workers is low. Having a person-centred perspective requires a broad understanding of individual capabilities, values, interests, and the environmental demands placed on individuals when they engage in occupations as described by the person-environment-occupation-performance model [49]. There is therefore a difficult balance between being flexible and person-centred while also having some form of standardised SP that is comparable in research across contexts.

### Study strengths and limitations

To ensure rigour, the procedure during data collection and quality assessment in this study followed the PRISMA guidelines [23]. A research librarian from the university library contributed to the development of search terms and offered guidance for the search process in order to find an optimal balance for finding and selecting a manageable number of articles. The four included systematic reviews had some overlap in the articles included; six empirical articles were included in two reviews and one empirical article was included in three reviews. The research group evaluated the reviews and considered them to be sufficiently varied to be included. A limitation was that the studies included participants that were younger than 65, as the amount of studies that only included older adults was limited. One of the study’s strengths was the research team’s wide range of professional backgrounds of occupational therapy, nursing, epidemiology and ethnology, which helped provide analyses from different perspectives. The heterogeneity of the included studies made it possible to examine a multitude of SP programmes even though the designs and methods of the studies varied. As nearly all the included studies were conducted within the context of the UK, future studies are highly needed to evaluate the impact in other national contexts, as well as include analysis on different socio-economic factors. Few articles reported on the attrition rate and all included studies used a per-protocol analysis, even though dropouts might have a severe impact on outcomes [57,58]. We recommend that further evaluations should include intention-to-treat analysis.

## Conclusions

By way of narrative analysis, this study identified three critical components, assessment before referral, matching participants with activities, and individualised support from link workers. These are suggested to be useful in the future development of social prescribing programmes when emphasizing or focusing on the impact of loneliness, health, and well-being. As such, a standardized procedure designed for broader implementation of social prescribing needs to leave room for individual tailoring while still following the core aspects of the programme. We recommend that future studies focus on different contexts and groups in society as well as include ITT-analysis to avoid attrition bias in the evaluations.

## Supplementary Material

Supplemental Material
