# Supplementary material for: Critical components of social prescribing programmes with a focus on older adults - a systematic review
Source: Scand J Prim Health Care. 2023 Jul 24;41(3):326–42. doi: 10.1080/02813432.2023.2237078 (PMC10478612; doi:10.1080/02813432.2023.2237078)
Supplement: Supplemental Material [file IPRI_A_2237078_SM0696.docx]

Appendix-document for Critical components of social prescribing programmes for older adults - A systematic review

1. Full search history
2. Prisma-flowchart
3. Descriptive table
4. Results table

Appendix 1, Full search history

Search terms: title or abstract: “social prescribing” OR “social prescription” OR “community referral” OR “community linkage” OR community connection”

| Search terms - Pubmed | Items found |
| --- | --- |
| Ti/ab “social prescribing” |  |
| OR ti/ab “social prescription” |  |
| OR ti/ab “community referral” |  |
| OR ti/ab “community linkage” |  |
| OR ti/ab “community connection” | 302 |

| Search terms - Medline | Items found |
| --- | --- |
| “social prescribing” |  |
| OR “social prescription” |  |
| OR “community referral” |  |
| OR “community linkage” |  |
| OR “community connection” | 304 |

| Search terms - PsychINFO | Items found |
| --- | --- |
| “social prescribing” |  |
| OR “social prescription” |  |
| OR “community referral” |  |
| OR “community linkage” |  |
| OR “community connection” | 201 |

| Search terms - CINAHL | Items found |
| --- | --- |
| “social prescribing” |  |
| OR “social prescription” |  |
| OR “community referral” |  |
| OR “community linkage” |  |
| OR “community connection” | 271 |

| Search terms - SOCIndex | Items found |
| --- | --- |
| “social prescribing” |  |
| OR “social prescription” |  |
| OR “community referral” |  |
| OR “community linkage” |  |
| OR “community connection” | 123 |

Appendix 2, Figure 1 Search strategy and outcomes


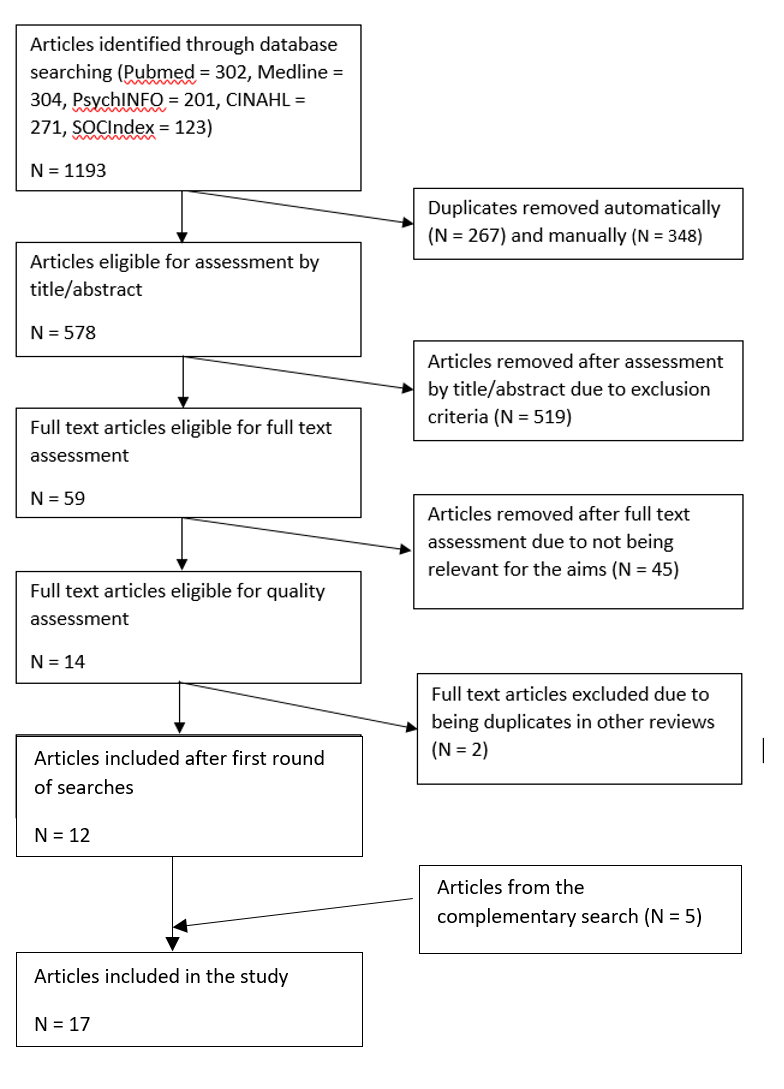


| Study  Appendix 3 – Table 1 Descriptive table | Method | Context | Participants | Outcome measures | Quality assessment | Attrition/ Adherence |
| --- | --- | --- | --- | --- | --- | --- |
| Bickerdicke 2017 | Systematic review. Nine databases were searched from 2000-2016 and analysed with narrative synthesis. 15 evaluations were included with varied methods and many forms of SP included. | All evaluations were from the UK. | No information reported | Warwick-Edinburgh Mental Well-being Scale (WEMWBS), Hospital Anxiety and Depression Scale (HADS), General Anxiety Disorder-7 (GAD-7), Patient Health  Questionnaire-9 (PHQ-9), Clinical Outcomes in  Routine Evaluation-Outcome Measure (CORE-OM), Work and Social Adjustment Scale (WSAS),  General Health Questionnaire (GHQ-12), Functional health with COOP/WONCA and some bespoke measuring tools.  Any measurement or reporting of attendance. Any reporting of costs.  Qualitative evaluations of patient experience and referrer experience. | Low risk of bias | Attrition rates were not reported.  Adherence to initial SP appointment ranged from 50-79% in the included studies. Adherence to social activity after referral ranged from 58-100% in the included studies. |
| Kilgariff-Foster 2015 | Literature review. 5 databases were searched and results analysed by narrative synthesis. 24 evaluations with varied methods and many forms of SP included. | All evaluations were from the UK. | The majority of participants were over 40 years old and female. Causes for referral was isolation due to unemployment or frequent GP attendees with inexplicable symptoms. | Any measures of health or well-being such as Anxiety and depression (HADS), Mental Well-Being (WEMWBS), General health (GHQ-12). Any other measures of impact.  Qualitative evaluation of participant experience. | Medium risk of bias | Attrition rates not reported.  Adherence to social activity only reported in one included study, 58%. |
| Peshceny 2019 | Systematic review. 11 databases were searched with relevant search terms and results analysed with narrative synthesis. 16 evaluations with varied methods and many different forms of SP programmes included. | All evaluations were from the UK. | Participants were described as older in five studies, referrals were for a mix of long-term conditions, frequent attenders of primary health care, psychosocial needs, and in five studies loneliness or social isolation. | Mental Well-Being (WEMWBS), Anxiety and depression (HADS), Measure Yourself Medical Outcome Profile (MYMOP), General health (GHQ-12), Functional health (COOP/WONCA), Friendship Scale.  Qualitative evaluation of patient experience. | Low risk of bias | Attrition rates not reported.  Adherence rates not reported. |
| Chatterjee 2018 | Systematised literature review. 12 databases were searched with relevant search terms. 40 evaluations were included, nine were SP and the rest a mix of other schemes. | All evaluations were from the UK. | 11 studies report including older adults. Majority of participants were included for some underlying or previous health issue. | Anxiety (GAD-7), Cost effectiveness (Quality Adjusted Life Years), Depression (PHQ-9), Functional Health (CO-OP/WONCA), Hospital admission (Hospital Episode Statistics HES) Mental health (GHQ), Mental well-being (WEMWBS), Physical activity (Timed Up and Go test), Psychological well-being (HADS), Quality of life (Delighted-Terrible faces), Social isolation (Social isolation), Social support (Duke-UNC Functional Social Support Questionnaire)  Qualitative evaluations of patient and professional experience. | Medium risk of bias | Attrition rates not reported.  Adherence rates not reported. |
| Elston 2019 | 12-month longitudinal pretest /posttest study. Questionnaire data were collected by healthcare staff from 126 participants during first and last meeting, healthcare use data were collected from IT systems. | Devon, UK, a rural area with a proportionally older population with some pockets of deprived areas. | Participants were over 50 years of age, 73% were female with two or more long-term conditions and many having assisted forms of living. | Well-being (Well-being Star), Activity (Patient Activation Measure), Mental Health & Well-being (Warwick-Edinburg Mental Well-Being Scale), Frailty (Rockwood Clinical Frailty Scale), health & social care use, health and social care costs. | Low risk of bias | 151 participants were referred to SP, 126 completed 12-week assessment, 86 completed full 12-month data.  Adherence rates not reported. |
| Morton 2015 | Pretest /posttest study. 136 participants filled out questionnaires at the beginning and end of their course. | Fife, UK | Participants were of an average age of 52 with older adults included, and many with ongoing mental health intervention/medication. | Anxiety and depression (Hospital Anxiety & Depression scale), Self-efficacy (General Self-Efficacy scale), Mental well-being (WEBWBS)  Anonymous feedback | Medium risk of bias | Attrition rates not reported.  Adherence rates not reported. |
| Pescheny 2020 | Pretest/ posttest study. Link workers collected data at first and last appointments from 63 participants. | Urban UK setting described as having a large ethnic minority with higher than average multiple deprivation. | Participants were of all ages, between 24 and 83, with a female majority and a majority outside employment. | Mental well-being (WEMWBS) | Medium risk of bias | 84.8% were lost to follow-up or did not engage after initial assessment.  Adherence rates not reported. |
| Kellezi 2019 | Mixed methods. Questionnaires were administered by healthcare workers at baseline (n=630) and after approx. 4 months (n=178). Semistructured interviews with 35 patients and staff, thematic analysis applied. | East Midlands, UK | Participants were from all ages, with an average age of 60.4 years. The SP programme was focused on people suffering from loneliness and chronic illness. | Questionnaires: social groups (custom), community belonging (custom), loneliness (UCLA loneliness scale), Health service use (custom).  Interviews: Participant experience of social connection and effects of SP programme. | Low risk of bias | Of the 630 participants, 178 participated in follow-up.  Adherence rates not reported. |
| Woodall 2018 | Mixed methods. Questionnaires were administered by link workers at first and last meetings, approx. six weeks, for 342 participants. 26 participants were interviewed and 17 link workers were recruited for a focus group discussion. These were analysed thematically. | A large city in northern England, UK | Participants were of a mean age of 53 years, ranging from 19-94, and a majority was female. | Questionnaires: well-being (WEMWBS), mental and physical health (EuroQol-5 Dimensions), social isolation and loneliness as well as the ability to manage long-term conditions (Campaign to End Loneliness Measurement).  Interviews: Experiences and outcomes, what works for whom and why, perspectives on the service. | Low risk of bias | Attrition rates not reported.  During this period, SP had 2250-3750 referrals, and they comment that the vast majority were lost to follow-up or did not engage in SP. |
| Heijnders 2018 | Interview study. Ten participants were purposefully chosen by link workers to include a variety of experiences from the programme. Results were analysed thematically. | Netherlands | Participants had a mean age of 69, withan even gender split. Patients had some psychosocial issue that was not explained by a medical condition. | What happens in the social prescription process?  What changes do people experience regarding social participation? | Medium risk of bias | Several participants had reduced activity attendance. |
| Payne 2020 | Interview study with a realist perspective. 17 participants were purposefully sampled from the programme. Semistructured interviews analysed by phenomenological analysis. | Sheffield, UK, that is described as a highly deprived area according to postcodes. | Participants were between 45-84 years old, majority female, mostly British. Many were referred for mental health reasons or for improving physical health. | Identify how participants perceived any benefits from SP. Emerging themes were tested against published qualitative findings. | Low risk of bias | Several participants had reduced progression through the programme. |
| Wildman 2019 | Interview study. Twenty-four participants with a variation of experiences were recruited. Semistructured interviews were analysed from a grounded theory perspective. | Newcastle upon Tyne, UK, described as an inner-city area with high socioeconomic deprivation | Participants were aged 40 to 74, and there was an even gender split, with mental health issues and multimorbidity being common. | Service users perspectives on link worker SP, with a focus on emergent narratives. | Low risk of bias | Several participants had reduced attendance to social activities. |
|  | Method | Context | Participants | Outcome measures | Quality assessment | Attrition/ Adherence |
| Bild 2022 | Systematic review. Four databases were searched from 2009-2019 and analysed with narrative synthesis. 77 articles were included with varied methods and many forms of SP included. | Not reported | The mean age of participants in each study was 50, with most included articles having a focus on older adults. | Any measures or qualitative results were described in themes of social connection; improvement in management of health and health status; improvement in mental health  and wellbeing; life enrichment; and link worker support. | Low risk of bias | Attrition rates not reported.  Adherence rates not reported. Lack of adherence measures in evaluations was reported as an issue. |
| Foster 2020 | Mixed methods.  Qualitative interviews with service-users, volunteers and link  workers and quantitative analysis of routinely collected data from 2017-2019 as well as additional data collected at 3 month follow-up. | The study included programs from a wide range of setting within the UK | The sample size was 10643 with 66% being female, 70% being white british, 65% lived alone, 80% were over 50 years of age, 50% experiencing health issues and 25% experiencing mobility issues. | Loneliness measured by UCLA Loneliness scale  Interviews focused on the experience and impact of the  support, service delivery and sustainability. | Low risk of bias | Attrition rates not reported, issues with collecting data mentioned.  Adherence rates not reported. |
| Kiely 2022 | Systematic review.  Eleven databases were searched up to 2021 and analysed with narrative synthesis. 8 articles were included with varied methods and many forms of SP included. | Primary health care and community contexts within the UK and USA. Two articles specifically mention being in a economically deprived area. | 6500 participants across the articles, majority female, age range from 29 -75. | Primary: Health-related quality of life (HRQoL), as measured by a validated instrument (EQ-5D, SF-12). Mental health outcomes, as measured by a validated instrument (HADS-A) for screening for mental health conditions.  Secondary:  Patient-reported outcomes on social-connectedness or isolation, self-rated health, patient experience of care, treatment burden, self-management behaviour and self-efficacy. Physical activity and function included measures of  physical activity (self-reported or objectively measured), physical function, activities of daily living. Health service utilisation measured via primary care or hospital records or self-reported. Any physical health data reported and any cost data or social return on investment data. | Low risk of bias | Attrition rates not reported.  Adherence rates not reported. |
| Kim 2020 | Pretest/ posttest study. Using the PRECEDE-PROCEED method, 10 participants were followed over a 10-week period. | Rural area within South Korea. | 10 participants, all female, mean age of 84, all had atleast one chronic disease, 7 participants were illiterate, 9 were living alone. | Depression (GDS-Korean), Loneliness (UCLA-Loneliness scale), Social participation attitude, Self-efficacy (GSE), Self-esteem (Rosenberg self-esteem scale) | Medium risk of bias | 16 participants, of which 10 participated the full 10 weeks and were included in the study.  All 10 adhered to atleast one activity, with some adhering to all three program activities. |
| Reinhardt 2021 | Systematic review.  Nine databases were searched from 2000 to 2019 and nine articles were synthesised, with highly heterogeneous studies and social prescribing programs. | All included studies were from the UK | 12359 participants plus approx. 9000 in one study. Participants age range from 16-85. | Loneliness (UCLA-loneliness scale, Adult Social Scale and Public Health Outcome, Hawthorne friendship scale) | Low risk of bias | Attrition only reported in one included article, from 254 at pretest to 215 at posttest.  Adherence not reported. |

| Components Studies  Appendix 4, Table 2 – Results table | **Assessment before prescription** | **Matching participants with activities** | **Link worker support** | Outcomes |
| --- | --- | --- | --- | --- |
| Bickerdicke 2017  Systematic review | In all studies, link worker met with participants to discuss their needs. | Focus on a wide range of community activities. Patient-centred focus not reported. | Link worker (LW) support in general was not reported. Some studies reported link workers having good local networks. Participants could receive support with navigating welfare programmes.  5 of 15 studies reported LW training, 7 did not have training, 3 did not report. | Health and well-being generally improved in quantitative measures, but authors recommend caution due to poor quality of included studies.  Patient experiences reflected reduced loneliness and social isolation, improvement in mental and physical health. Experiences also raised the importance of confidence, successful matching, overcoming barriers, informed referral, and clear communication between actors. |
| Kilgariff-Foster 2015  Systematic review | One study described 40-90-minute appointment to identify needs and appropriate activities. Assessment was not reported in other studies. | Focus on a wide range of community activities, with one study described finding appropriate activities, but no other patient-centred focus reported. | LW support was in-person meetings. One study described LW accompanying patients to activities, with patient receiving increased support early. Another study described a small number of follow-up appointments, average was two.  LW received training in all included studies. | Reduced anxiety (p<0.002), improvement in well-being, reduction in symptoms and attaining goals, but authors recommended caution due to poor quality.  Patient experience highlighted self-efficacy. Too long wait times resulted in patient not engaging. |
| Pescheny 2019  Systematic review | All included studies had individual assessment to identify non-medical needs of service users, sometimes using tools or motivational interviewing. | Focus on a wide range of community activities, with most studies describing some form of patient-centred focus. | Link worker support was in-person meetings in all studies. Support was described as personalized and continuous. LW accompanied patients to activity in one study, two studies described increased support early with a patient-centred focus. Participants could receive support with navigating welfare programmes.  LW training was partially reported. | No significant results for mental health. Results for well-being, general health, function, and loneliness was mixed. Patients also felt reduced social isolation in qualitative studies.  Patient experience highlighted support from link workers as central for behavioural change, attendance, building confidence, and overcoming barriers in their daily lives. Mastering skills made participants continue. |
| Chatterjee 2018  Systematised review | Not reported | Partial focus on a wide range of community activities. Patient-centred focus not reported | LW support was in-person meetings. Results include 3 studies with “supported referral” that was described as helping overcome barriers and offering moral support depending on need. Signposting schemes could offer guidance to welfare programmes.  LW training was partially reported. | Reduced anxiety, depression, loneliness, and social isolation. Improved mental well-being and physical health. Improved confidence and self-efficacy. Link workers with local knowledge an important facilitator, with limited choices and lack of economical support important barriers.  No statistical results were given, quantitative and qualitative outcomes were reported together, and authors recommended caution due to poor quality. |
| Elston 2019 12-month pretest/ posttest | Initial 30 min meeting to determine light or “holistic” approach. Holistic approach consisted of using multiple tools over several meetings. | Focus on a wide range of community activities, with a focus on understanding what matters to participants and setting goals for living well. | LW support was in-person and by phone or home visits when needed. Support consisted of coaching and practical support over 12 weeks, also support for navigating welfare systems.  LW received training in goal-setting, using tools, using a strength-based focus, co-producing a plan, and managing risk. Key skills included listening skills, emotional support, advice, practical assistance, and coaching. | Improved mental well-being (P = 0.000), well-being (P = 0.000), patient activation (45/81, 55.6%), reduced frailty (4.6%).  Link worker improved continuity of care and offered support for both patients and carers during difficult times. Some patients had sudden deteriorating health that affected outcomes. |
| Morton 2015  Pretest/ posttest | Not reported | Only a few courses offered as part of the SP programme (Meditation, Painting, Photography, Jewellery, Arts & Crafts, and Pottery). Patient-centred focus not reported | LW support was in-person meetings as part of the courses offered. Participants received no support outside courses.  LW received training in working with mental health, such as identifying and supporting someone with anxiety. | Reduction in depression and anxiety (P < 0.001), improved mental well-being (P < 0.001), and improved self-efficacy (P < 0.001). Majority of participants also received therapy or medical treatment during the intervention. |
| Kellezi 2019  Mixed methods | Initial one-hour needs assessment with healthcare personnel who then referred to LW. | Focus on a wide range of community activities, with a patient-centred focus | LW support was in-person meetings over 8 weeks, with programme length dependent on the pathway. LW regularly checked participant’s progress and could accompany participant to first activity if needed.  LW training not reported | Feelings of group membership increased (p=0,022), which was a positive predictor for community belonging (p=0.01), which in turn was a negative predictor for loneliness (p=0.0001) that positively predicted reduced primary healthcare use (p=0.002).  GP experience reflected need for holistic interventions, and that in the current medical model GPs exacerbate rather than help with poor social health. LW experience reflected on patients not knowing what was around them and the importance of reconnecting with the community. Patient experience positively noted the increased amount of time they had to discuss their problems and the tailored, encouraging support they received. Patients reported increased confidence and described LW support as vital, especially when the LW accompanied them to their first activity, where being positively welcomed was crucial. The LW support was essential in connecting with others and sustaining meaningful connections in groups.  SP could help others besides the patient, causing a “ripple effect”. Important to have a feedback loop. |
| Woodall 2018  Mixed method | Needs assessment could be short and over the phone, or longer with in-person meetings. | Focus on a wide range of community activities, with a focus on exploring social support needs. | LW support was in-person meetings and by phone when needed. Target limit was 6 sessions, but many exited within 16 weeks, with an explicit statement to avoid participants becoming dependent on the service. The SP service did not offer much support, but activities could be very supportive. Could offer finance/debt advice  LW received training. | Improved well-being (p < 0.001) with a negative relationship with age (p < 0.001), especially under age 50 (p = 0.02). This was supported by interviews.  Reduced depression & anxiety (p < 0.001), improved self-rated health (p < 0.001), and small improvement to social network scores (p < 0.001, d = 0.35). Interviews reflected increased social connectedness due to finding good matches between patients and activities. This led to increased feelings of confidence and purpose.  Sharing experiences in social settings, having skilled link workers, flexible support, and a thriving community were seen as important for success, particularly in engaging men. |
| Pescheny 2020  Before/after study | Individual assessment with motivational interviewing to identify non-medical needs. | Focus on a wide range of community activities. Patient-centred focus not reported. | LW support was in-person meetings, with support tailored according to person’s needs and continuous personalised support offered.  LW training not reported. | Statistically improved mental well-being (P < 0.0001), but the results were not clinically significant.  The authors reported LW turnover, fragmented service delivery, language barriers, and difficulties in completing questionnaire as possible reasons for the high loss to follow-up. |
| Payne 2020  Interview study | Participants were triaged by phone or in person. | Focus on a wide range of community activities, with a focus on personalised linking to activities. | LW support was in-person and by phone, with LW accompanying participants to activities when needed. Programme length was between six months and five years.  LW training not reported. | Improved confidence the central positive outcome in all themes.  Five central themes for a successful intervention were discussed; **Receiving professional support** to overcome practical barriers and improving confidence, **Engagement with others** in purposeful and enjoyable activities that provided routine, **Learning new skills** improved confidence and reinforced a shared experience, **Changing perceptions** provided improved confidence and an appreciation of personal strengths, and **Developing a positive outlook,** where improved confidence and independence motivated participants to pursue new goals and have optimism for the future.  Other healthcare needs were an important reported barrier for participation among those that did not progress as expected. |
| Heijnders 2018  Interview study | Intake session with strengths-based approach to evaluate participant’s life holistically in order to find sources of positive strength and also possible barriers to participation. | Focus on a wide range of community activities, with a step-by-step approach that focused on what the participant enjoys doing, and with encouragement to choose activities that promote social health. | LW support was in-person meetings and by phone, or home visits when needed. LW also registered for activities together with the participant and monitored progress by phone.  LW received training. | The authors summarized outcomes in which participants mostly felt healthier, became more self-reliant, and regained perspective and control over their lives.  Five themes emerged in the study: **Life events** highlighted the sudden changes that caused the participants current situation, such as loss of spouse or retirement. **Referral and intake process** highlighted the major obstacle of starting something new alone, and the personalised service with follow-ups encouraged adherence. **Strength and responsibility** highlighted the importance of activities reflecting participants needs, interests, and hobbies as well as finding their own solutions to problems. **Self-reliance** highlighted the need for strong incentives and support from the LW and the community for continued participation. **Social activation** also highlighted the importance of matching interests and connecting with others. |
| Wildman 2019  Interview study | Evaluation by motivational interviewing & proprietary tool to find life areas that the participant wanted to improve. | Focus on a wide range of community activities, with LW helping patients identify personalised and achievable goals. | LW support was in-person meetings and by phone, or home visits when needed. Programme length was on average two years, with option to go longer. LW had regular check-ups to monitor progress towards participants own goals. LW would accompany participant to activity when needed. Programme could address housing, debt, and welfare needs.  LW received training in behavioural change methods. | Themes that emerged were**: Importance of the LW-participant relationship** with the personalised approach seen positively. The LW was essential in building confidence as was their wide knowledge of community services. **Making and maintaining progress and long-term condition management** highlighted the participant’s growing confidence as central for improvement, and the long-term focus of the programme facilitated continued self-regulation. **Setbacks and barriers** highlighted the difficulty of maintaining change over time, with health-related problems being the most common cause of relapse, or physical barriers such as lengthy or costly travel, unsuitable scheduling, an unsafe location, language barriers, or cultural inappropriateness. LW turnover and poor continuity was also reported as detrimental. **Fluctuating levels of engagement** highlighted that contact with SP service declined naturally as participants engaged in activities, but for some, the two-year time constraint was seen as too short, and they needed much more long-term support. |
| Bild 2022 Systematic review | Programs included a wide range of different assessments as well as no assessments at all. | Most included programs had a focus on specific activities, such as arts & crafs or walking groups. Some programs had a focus on a wide range of community activities. | LW support included a wide range, from “group leaders” to trained LW-professionals. Some programs were open ended, with others having a clear structure in time and amount of LW-meetings.  LW training varied significantly in the different programs. | Improved social connection, including a sense of group connection and reduced feelings of loneliness.  Improved mental health and wellbeing, although authors recommend caution as there is some indication that those with poor mental health and wellbeing are not always included in the results of evaluations due to attrition/adherence.  Increased physical activity, increased sense of purpose, independence, and confidence due to new meaningful activities. |
| Foster 2020 Mixed method | Link-workers and volunteers performed an needs assessment. | Focus on developing confidence in order for participants to access a wide range of community activities with a person-centred focus. | LW support amount and length was tailored according to individual´s needs, with volunteers offering further support such as following participants to activities. | Reduced loneliness, favourable return on investment.  Qualitative experiences reflect improved self-esteem, confidence and wellbeing. |
| Kiely 2022  Systematic review | The programs describe face-to-face needs assessment by link-worker, action planning and motivational interviewing, mapping resources, | Referral activities were tailored to the individual with a focus on a wide range of community activities. | Link-worker support varied from 1 meeting to 2 years of ongoing support, with specific type of support not reported. | No evidence for improved health-related quality of life or mental health. no evidence for effectiveness in improving social support, physical function and activities, or primary healthcare utilisation, though there was a suggestion from two studies that interventions led to improved self-rated health and two others reported higher patient ratings for quality care.  The certainty of the evidence is low or very low overall due to risk of bias, heterogeneity among studies, inconsistency and imprecision. |
| Kim 2020 Pretest/  posttest | Face-to face health evaluation with public health doctor | Referral activities were a music group, a self-help group and a gardening group. | Coordinators and volunteers held the program activities, level of support in our outside programs was not reported. | Reduced loneliness and depression, improved social participation attitude and self-esteem. |
| Reinhardt 2021 | Assessment not clearly reported | Included articles ranged from a focus on individually tailored community activities to a more specific activity such as prescribed museums. | Support varied from simple signposting to long-term individualised support from link-workers. | Reduced loneliness and use of services especially among those aged under 60. |
